# Prevalence of Hearing Loss Among US Adolescents

**DOI:** 10.1001/jamanetworkopen.2024.58854

**Published:** 2025-02-10

**Authors:** Harry C. Wu, Michel Neeff, Frank R. Lin

**Affiliations:** 1 Department of Otolaryngology–Head and Neck Surgery, Auckland City Hospital, Auckland, New Zealand; 2 Department of Pediatric Otolaryngology–Head and Neck Surgery, Starship Hospital, Auckland, New Zealand; 3 Department of Otolaryngology–Head and Neck Surgery, Johns Hopkins School of Medicine, Baltimore, Maryland

## Abstract

This cross-sectional study of national survey data from US adolescents examines changes in rates of reported hearing loss, including associations with sociodemographic characteristics.

## Introduction

The prevalence of hearing loss among adolescents is an important public health issue. Even a mild degree of hearing loss can detrimentally impact cognitive development, academic achievement, and social engagement.^1,2^

Hearing loss in adolescents has been ascribed to genetic, congenital, traumatic, and infectious causes.^3^ A 2010 study from Shargorodsky et al^4^ and a 2017 study from Su et al^5^ both noted a fluctuation in hearing loss prevalence in US adolescents aged 12 to 19 years, increasing from 14.9% in 1988 to 19.5% in 2005, then decreasing to 15.2% by 2010. These trends suggest that additional factors—perhaps related to medical advancements like the increased uptake of the pneumococcal conjugate vaccine in children, changing demographic factors, and modern lifestyles with noise exposure and the prevalent use of personal audio devices—may affect hearing loss prevalence.^6^

Understanding and characterizing the shifts in hearing loss prevalence among adolescents is crucial for shaping effective public health strategies. Our investigation examines National Health and Nutrition Examination Suvery (NHANES) data from 2005 to 2020 to assess the temporal changes in the prevalence of hearing loss in this population.

## Methods

NHANES is a continuously conducted survey that evaluates the health and nutritional status of the civilian noninstitutionalized population in the US. It employs a sampling method which results in a nationally representative sample. The study was conducted in adherence to the Strengthening the Reporting of Observational Studies in Epidemiology (STROBE) reporting guideline for cross-sectional studies. Institutional review board approval was not required for analysis of deidentified publicly available data per the Common Rule.

We analyzed data from the 2005-2006, 2007-2008, 2009-2010 and 2017-2020 cycles. Our analytic cohort comprised adolescents aged 12 to 19 years in each cycle with available audiometric and covariate data. Audiometry was conducted using established NHANES protocols in sound attenuating booths.

In line with previous analyses, low-frequency pure tone average (LPTA) was calculated using pure-tone thresholds at 0.5, 1, and 2 kHz. High-frequency pure tone average (HPTA) was calculated from the pure-tone thresholds at 3, 4, 6, and 8 kHz. Hearing loss is defined as either LPTA or HPTA greater than 15 dB and 20 dB in the worse ear.^4,5^ In an effort to standardize hearing loss definitions, analyses were also conducted in accordance with the World Health Organization hearing classification, defining hearing across speech frequencies PTA of 0.5, 1, 2, 4 kHz (PTA4).

In separate analyses aimed at identifying potential risk factors for hearing loss, multivariable logistic regression was conducted using all available data from 2005 to 2020 for a representative cohort of 12-to-19-year-olds. This analysis was used to evaluate the association of demographic factors and hearing-related variables with the odds of hearing loss. The models were controlled for age, sex, ethnicity, poverty-to-income ratio, and recurrent ear infections as known demographic and hearing covariables.

To account for the complex sampling design of NHANES, our analysis incorporated recommended sample weights to ensure the analysis is generalizable to the US population. Stata version 16 (StataCorp) was used for data analysis (eMethods in Supplement 1).

## Results

A total of 5426 adolescents were analyzed in this study with a mean (SD) age of 15.5 (0.04) years. Cohorts had similar age and sex prevalences and have grown more diverse from 2005-2006 to 2017-2020 (Table 1). Hearing loss prevalence decreased from 19.5% (95% CI, 15.7-23.9) in 2005-2006 to 10.9% (95% CI, 9.3-12.7) in 2017-2020. In the weighted logistic regression models of combined 2005-2020 data, we found that female adolescents (odds ratio [OR], 0.78; 95% CI, 0.66-0.93), adolescents identifying as non-Hispanic Black (OR, 0.74; 95% CI, 0.58-0.95) or other race or ethnicity (OR, 0.64; 95% CI, 0.42-0.97), and adolescents living in households above the poverty threshold (OR, 0.73; 95% CI, 0.57-0.92) were associated with reduced odds of hearing loss (Table 2). Adolescents experiencing 3 or more ear infections had greater odds of hearing loss (OR, 1.69; 95% CI, 1.28-2.24).

**Table 1.** Demographic Characteristics and Prevalence of Hearing Loss Among Adolescents Aged 12 to 19 Years in Study Cohort^a^

Characteristics	Weighted prevalence (95% CI)			
	NHANES 2005-2006 (n = 1773)	NHANES 2007-2008 (n = 1008)	NHANES 2009-2010 (n = 1116)	NHANES 2017-2020 (n = 1529)
Weighted Count, No.	26 187 670	27 250 257	27 242 403	28 412 777
Age, y				
12-13	23.5 (20.1-27.4)	20.0 (16.7-23.6)	23.4 (20.9-26.3)	25.7 (23.2-28.3)
14-15	26.8 (24.3-29.3)	28.0 (24.5-31.8)	26.9 (24.5-31.8)	23.6 (20.4-27.0)
16-17	26.9 (23.9-30.2)	26.9 (23.8-30.1)	27.3 (24.6-30.2)	28.3 (26.5-30.2)
18-19	22.8 (18.8-27.3)	25.2 (22.6-28.0)	22.8 (19.2-25.9)	22.5 (20.3-24.9)
Sex				
Male	51.2 (48.0-54.5)	51.8 (49.0-55.6)	51.5 (47.6-55.4)	50.9 (45.5-52.0)
Female	48.8 (45.6-51.9)	48.2 (44.4-52.0)	48.5 (44.6-52.4)	49.2 (44.5-53.9)
Race^b^				
Hispanic or Mexican	16.2 (13.5-19.3)	18.7 (13.6-25.3)	19.7 (14.0-27.4)	24.0 (18.6-30.4)
Non-Hispanic Black	14.3 (9.5-21.0)	14.9 (11.1-19.8)	14.3 12.0-16.9)	12.4 (9.1-16.8)
Non-Hispanic White	62.2 (55.5-68.4)	61.6 (53.6-68.9)	58.2 (50.6-65.4)	52.4 (45.6-59.0)
Other or mixed	7.4 (4.9-11.0)	4.8 (2.8-8.0)	7.8 (5.7-10.5)	10.9 (9.0-13.0)
Poverty income ratio ≥1^c^	82.2 (78.5-85.3)	78.4 (73.5-82.5)	77.4 (72.4-81.8)	78.9 (75.0-82.4)
History of >3 ear infections^d^	38.7 (34.2-43.4)	39.2 (33.8-44.9)	34.3 (31.3-37.4)	29.3 (25.4-33.5)
**Prevalence of hearing loss**				
Historical definitions				
Worse ear high frequency PTA^e^	16.3 (13.4-19.8)	17.7 (13.5-22.9)	10.8 (9.3-12.7)	9.2 (7.5-11.1)
Worse ear low frequency PTA^f^	8.9 (6.2-12.5)	9.4 (6.8-12.9)	7.5 (5.9-9.6)	5.1 (4.2-6.1)
Worse ear high or low frequency PTA	19.5 (15.7-23.9)	20.5 (16.3-25.5)	14.2 (11.8-17.0)	10.9 (9.3-12.7)
Better ear high frequency PTA	3.9 (2.8-5.3)	4.3 (3.3-5.8)	2.8 (1.7-4.6)	2.0 (1.3-2.9)
Better ear low frequency PTA	2.3 (1.7-3.1)	3.6 (2.0-6.3)	1.8 (1.1-2.9)	1.3 (0.7-2.3)
Better ear high or low frequency PTA	5.2 (3.9-7.0)	6.2 (4.3-8.8)	4.1 (2.9-5.9)	2.5 (1.7-3.6)
World report on hearing definition^g^				
Worse ear PTA4	7.0 (5.2-9.3)	8.8 (6.3-12.2)	6.1 (4.6-8.2)	4.6 (3.7-5.8)
Better ear PTA4	2.7 (2.0-3.6)	3.9 (2.5-6.2)	1.7 (1.0-2.9)	1.5 (0.9-2.3)
**Mild or worse hearing loss (≥20 dB)**				
Historical definitions				
Worse ear high frequency PTA^e^	8.5 (6.3-11.3)	8.0 (5.9-10.7)	5.1 (4.1-6.4)	4.3 (3.5-5.3)
Worse ear low frequency PTA^f^	4.9 (3.7-6.6)	4.8 (3.5-6.6)	4.3 (3.2.-5.8)	2.2 (1.5-3.3)
Worse ear high or low frequency PTA	10.1 (7.8-13.0)	9.6 (7.6-12.1)	7.2 (5.7-9.2)	5.3 (4.3-6.6)
Better ear high frequency PTA	1.3 (0.7-2.6)	1.7 (0.1-2.9)	0.7 (0.3-1.6)	1.0 (0.5-1.8)
Better ear low frequency PTA	1.0 (0.5-1.8)	0.6 (0.2-1.9)	0.1 (0.0-0.5)	0.6 (0.3-1.2)
Better ear high or low frequency PTA	1.9 (1.2-3.1)	1.9 (1.1-3.1)	0.8 (0.3-1.7)	1.2 (0.7-2.0)
World report on hearing definition				
Worse ear PTA4	2.4 (1.5-3.6)	3.6 (2.3-5.7)	2.1 (1.4-3.2)	1.7 (1.1-2.4)
Better ear PTA4	0.5 (0.3-0.9)	1.4 (0.7-2.6)	0.4 (0.1-1.2)	0.7 (0.4-1.3)

Abbreviations: NHANES, National Health and Nutritional Examination Survey; PTA, pure tone average.

^a^ Composite of NHANES 2017-2018 and prepandemic 2019 to early 2020 data.

^b^ Participants were asked to choose from predefined categories: non-Hispanic White, non-Hispanic Black, Mexican American, other Hispanic, and other race (including multiracial). Hispanic or Mexican is a combination of the Mexican American and other Hispanic self-reported categories.

^c^ Poverty income ratio is the total family income divided by the poverty threshold, as determined by the US Census Bureau. Less than 1 indicates that the participants’ family income is below the US poverty threshold.

^d^ Coded based on self-report of 3 or more episodes of acute otitis media. The possible responses were recorded as yes, no, or missing.

^e^ High frequency PTA defined by the average pure-tone average of (3 kHz, 4 kHz, 6 kHz, 8 kHz).

^f^ Low frequency PTA defined by average pure-tone average of (500 Hz, 1- kHz, 2 kHz).

^g^ 2021 World report on hearing standardized criteria across 4 frequency pure-tone average (PTA4) across (500 Hz, 1 kHz, 2 kHz, 4 kHz).

**Table 2.** Weighted Multivariable Logistic Regression for Hearing Loss 15 dB or Higher, 2005-2020^a^^,^^b^

Hearing covariable	Odds of hearing loss (95% CI)^c^
Age, y	
12-13	1 [Reference]
14-15	1.18 (0.87-1.62)
16-17	1.26 (0.94-1.69)
18-19	1.32 (0.94-1.84)
Sex	
Male	1 [Reference]
Female	0.78 (0.66-0.93)
Race and ethnicity	
Non-Hispanic White	1 [Reference]
Non-Hispanic Black	0.74 (0.58-0.95)
Hispanic or Mexican	1.03 (0.79-1.33)
Other or mixed	0.64 (0.42-0.97)
PIR	
<1	1 [Reference]
≥1	0.73 (0.57-0.92)
Recurrent ear infections	
<3	1 [Reference]
≥3	1.69 (1.28-2.24)

Abbreviations: NHANES, National Health and Nutritional Examination Survey; PIR, poverty income ratio.

^a^ Model controlling for demographic and hearing covariables of age, sex, ethnicity, PIR, and having had more than 3 or more ear infections.

^b^ Appended data from the 2005-2006, 2007-2008, 2009-2010, and 2017-2020 NHANES cycles. Data weighted to be nationally representative.

^c^ Hearing loss defined as pure tone average of either high (3, 4, 6, 8 kHz) or low (0.5, 1, 2 kHz) frequency loss in the worse hearing ear >15 dB.

## Discussion

In a nationally representative study, adolescents were less likely to have audiometric hearing loss in 2017-2020 compared with 2005-2006. These findings are consistent with broader research trends, which observed a rise in hearing loss rates from 1988-2008, followed by a subsequent decrease up to 2009-2010.^5^

While the exact drivers behind this lower prevalence remain uncertain, it could be in part attributed to the reduction in the prevalence of recurrent ear infections and a decline in demographic factors known to be associated with hearing loss (eg, White race as a proxy for lighter skin color and poverty). A limitation of our analyses is the lack of available data on other important factors including environmental noise exposure and the use of headphones that are associated with hearing loss.

## Notes

Supplement 1.
eMethods.

Supplement 2.
Data Sharing Statement
