# Supplementary material for: Prevalence of Hearing Loss Among US Adolescents
Source: JAMA Netw Open. 2025 Feb 10;8(2):e2458854. doi: 10.1001/jamanetworkopen.2024.58854 (PMC11811794; doi:10.1001/jamanetworkopen.2024.58854)
Supplement: Supplement 1. — eMethods. [file jamanetwopen-e2458854-s001.pdf]

Supplemental Online Content

Wu HC, Neeff M, Lin FR. Prevalence of hearing loss among US adolescents. *JAMA Netw Open.* 2025;8(2):e2458854. doi:10.1001/jamanetworkopen.2024.58854

eMethods

This supplemental material has been provided by the authors to give readers additional information about their work.

© 2025 Wu HC et al. *JAMA Network Open*.

eMethods

NHANES is a continuously conducted health survey conducted by the Centers for Disease control (CDC) that evaluates the health and nutritional status of the civilian non-institutionalized population in the United States. It employs a sampling method which results in a nationally representative sample. NHANES data released in 2-year cycles. Each cycle of data consists of questionnaires and a standardized health examination.

Our analytic cohort comprised adolescents aged 12-19 years in each cycle with available audiometric and covariate data from the 2005-2006, 2007-2008, 2009-2010 and 2017-2020 cycles. Audiometry in 12–19-year-olds was not conducted in NHANES cycles between 2010-2017.

Audiometric measures:

Audiometry was conducted in sound-attenuating booths in the mobile examination centers in line with established NHANES protocols by a trained examiner. The equipment and testing protocol remained unchanged across the time period.

Air-conduction thresholds for each ear were measured using standardized audiometric headphones, covering frequencies from 0.5, 1, 2, 3, 4, 6, and 8 kHz, with intensity levels ranging from −10 dB to 120 dB. To ensure the reliability of the participants' responses, the 1 kHz frequency was tested twice in each ear. Pure-tone audiograms were not accepted if there was a 10-dB or greater difference between the 1-kHz test-retest thresholds.

© 2025 Wu HC et al. *JAMA Network Open*.

A retesting protocol was implemented to prevent signal crossover between ears by bone conduction. If there was a threshold difference of 25 dB at 0.5 and 1 kHz, or a 40 dB difference at higher frequencies between the ears, retesting was conducted using insert earphones. The retest values were then utilized as the threshold. Adolescents who had cochlear implants or were unable to tolerate headphones were excluded from the study.

Hearing Loss Definitions

In line with previous analyses, low-frequency pure tone average (LPTA) was calculated using the average of pure-tone thresholds at 0.5, 1, and 2 kHz. High-frequency pure tone average (HPTA) was calculated from the average of pure-tone thresholds at 3, 4, 6, and 8 kHz. HL is defined as either LPTA or HPTA >15dB in the worse ear.

In an effort to standardize HL definitions across studies, analyses were also conducted in accordance with the 2021 WHO world report on hearing classification, defining hearing across speech frequencies PTA of 0.5, 1, 2, 4 kHz (PTA4).

Hearing related covariates

Age was categorized into four groups: 12-13, 14-15, 16-17, and 18-19 years old. Race-ethnicity were grouped as non-Hispanic Black, non-Hispanic White, Hispanic (as a composite of Mexican American and Other Hispanic) and Other based on NHANES self-report groupings.

Participants were asked if they had experienced three or more episodes of acute otitis media.

© 2025 Wu HC et al. *JAMA Network Open*.

The poverty-income ratio (PIR) was calculated by dividing the total family income by the poverty threshold for the interview year, as defined by the US Bureau of the Census. PIR values less than 1 indicated income below the poverty threshold, while values of 1.00 or greater indicated income at or above the poverty level.

Definitions relating to noise exposure had changed between 2005-2010 and 2017-2020 cycles of NHANES so were excluded from the analyses.

In separate analyses aimed at identifying potential risk factors for HL, multivariable logistic regression was conducted using all available data from 2005 to 2020 for a representative aggregate sample of 12-19-year-olds. This analysis was used to evaluate the relationship between demographic factors and hearing-related variables with the odds of hearing loss. The models controlled for covariables of age, sex, ethnicity, poverty-to-income ratio, and recurrent ear infections

The study was conducted in adherence to the STROBE criteria for observational research.

Institutional review board approval was not required for analysis of de-identified publicly available data.

Statistical Analyses

© 2025 Wu HC et al. *JAMA Network Open*.

To account for the complex sampling design of NHANES, our analysis incorporated published sample weights to ensure the analysis is generalizable to the US population.

Stata, version 16 (StataCorp) was used for data analysis.

© 2025 Wu HC et al. *JAMA Network Open*.
