# Supplementary material for: Prevalence of Hearing Loss Among US Adolescents
Source: JAMA Netw Open. 2025 Feb 10;8(2):e2458854. doi: 10.1001/jamanetworkopen.2024.58854 (PMC11811794; doi:10.1001/jamanetworkopen.2024.58854)
Supplement: Supplement 2. — Data Sharing Statement [file jamanetwopen-e2458854-s002.pdf]

Data Sharing Statement

Data
Data available: No

Additional Information
Explanation for why data not available: NHANES data is widely available on public CDC repository with data dictionaries published online at
https://www.cdc.gov/nchs/nhanes/index.htm
